# The effect of redox agents on conformation and structure characterization of gluten protein: An extensive review

**DOI:** 10.1002/fsn3.1937

**Published:** 2020-10-15

**Authors:** Elahe Abedi, Kiana Pourmohammadi

**Affiliations:** ^1^ Department of Food Science and Technology College of Agriculture Fasa University Fasa Iran

**Keywords:** chemical modifications, conformation changes, gluten, physico‐chemical properties

## Abstract

Gluten protein as one of the plant resources is affected by redox agent. Chemical modifications by redox agent have myriad advantages mainly short reaction times, no requirement for specialized equipment, low cost, and highly clear modification impacts. The gluten network properties could be influenced through redox agents (oxidative and reducing agents) which are able to alter the strength of dough via different mechanisms for various purposes. The present review examined the impact of different redox compounds on gluten and its subunits based on their effects on their bonds and conformations and thus with their impacts on the physico‐chemical, morphological, and rheological properties of gluten and their subunits. This allows for the use of gluten for different of purposes in the food and nonfood industry.

## Graphical abstract

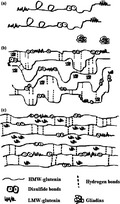

## 1 INTRODUCTION

**FIGURE 1:**
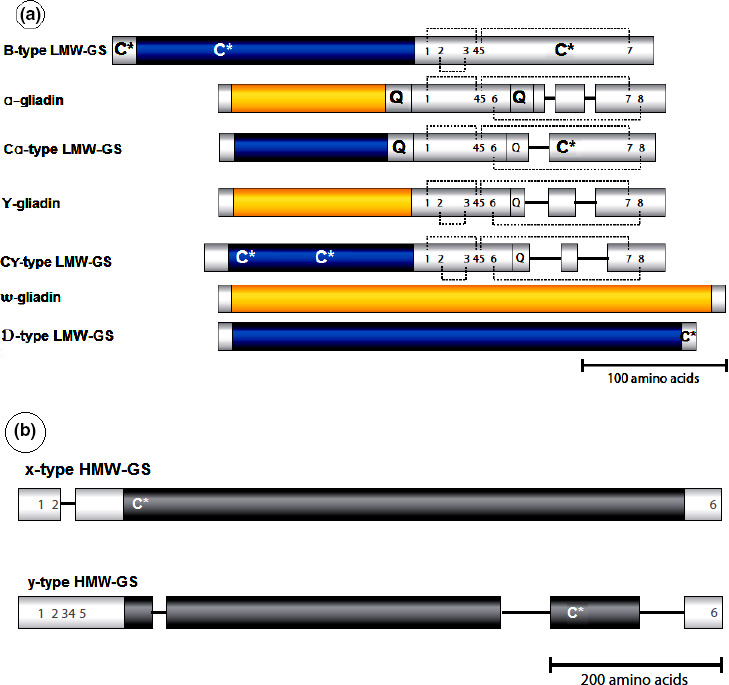
(a) α‐, γ‐, and ω‐gliadin structures (yellow) and B, C, and D LMW‐GS (blue). (b) Structure of x‐ and y‐type HMW‐GS. Repetitive domains are colored, while nonrepetitive domains are white. Positions of the conserved cysteine residues are indicated with Arabic numerals. Additional unconserved cysteine residues are indicated with C*. Intramolecular disulfide bonds are indicated by the gray dotted lines. Q represents a polyglutamine sequence. Similar polypeptide regions are aligned based on their amino acid sequences (Delcour et al., 2012)

Gluten proteins as a family of storage proteins obtained from the grains of wheat are comprised of gliadin fraction and glutenin fraction. Gliadin soluble in alcohol–water 70% ethanol has monomer structures with intramolecular disulfide (SS) bonds (except ω‐gliadins) containing fractions such as α/β (28,000–35,000, 28%–33%), γ‐gliadins (31,000–35,000, 23%–31%), ω_1,2_ (39,000–44,000; 4%–7%), and ω_5_ (49,000–55,000; 3%–6%) relied on their amino acid compositions, partial or complete amino acid sequences, and MWs (Figure 1; Table 1) (Abedi et al., 2018; Day et al., 2006; Majzoobi & Abedi, 2014; Majzoobi et al., 2012; Wieser, 2007). The glutenin subunit is insoluble in ethanol and generates intermolecular SS bonds, which is characterized into two types, namely HMW glutenin subunits (HMW‐GS) and LMW glutenin subunits (LMW‐GS) which contain MWs with an approximate proportion of (83,000–67,000–88,000, 10%) and (32,000–39,000, 19%–25%) of total gluten proteins, respectively (Joye et al., 2009a). LMW‐GS and HMW‐GS comprise of eight and eleven‐twelve cysteines, respectively (Delcour et al., 2012; Grosch & Wieser, 1999; Tatham et al., 1985; Wang et al., 2015). Covalent bonds, including disulfide bonds isopeptides, dehydroalanine (DHA), lanthioalanine (LAN), lysinoalanine (LAL), and dityrosine, and noncovalent, namely hydrogen, ionic, and hydrophobic bonds (Wieser, 2007), are crucial to gluten functionality under ambient conditions. Although gluten proteins contain few cysteine residues (~2% of the total amino acid composition), they are more susceptible to modification in the structure and function of gluten proteins (Wieser, 2007). Extensively discussed in the present review, three competitive reactions involving thiol (SH) groups impact the gluten network: (a) oxidation of free SH groups (polymerization), (b) reaction with “terminators” (blocking polymerization), and (c) SH/SS interchange reactions with low molecular weight SH compounds including glutathione (GSH) and gliadins that have an odd number of cysteines (depolymerization) (Joye et al., 2009a, 2009b).

**TABLE 1:** Characteristics of gliadins and glutenin

Proteins	Type	Molecular weight × 10^3^	Proportion (%)	Partial amino acid composition (%)					Cystein
				Q	P	F	Y	G	
Gliadins	ω_5_	49–55	3–6	56	20	9	9	1	0
	ω_1,2_	39–44	4–7	44	26	8	1	1	0
	α/ β	28–35	28–33	37	16	4	3	2	6
	γ	31–35	23–31	35	17	5	1	3	8
Glutenin	x‐HMW	83–88	4–9	37	13	0	6	19	4–5
	y‐HMW	67–74	3–4	36	11	0	5	18	7
	LMW	32–39	19–25	38	13	4	1	3	8

Note

Percentage according to total gluten proteins.

Number of cysteine residue.

Sourced from Grosch and Wieser (1999); Wieser (2007).

The network properties of gluten could be influenced through redox agents which are able to modify the strength of dough for various purpose via different mechanisms. Improved properties of dough are mainly ascribed to the features of the flour, the gluten nature (gliadin/glutenin ratio), composition of amino acid, the amount and type of redox agent (Table 2), and the breadmaking procedure. The present review examined the impact of different redox compounds on gluten and its subunits based on their effects on their bonds. This allows for the use of gluten for a variety of purposes in the food and nonfood industry.

**TABLE 2:** The effect some redox agents to functional, rheological of gluten

Type of acid	Object	Approach	Ref
Potassium iodate (0.82–2.47 µmol/g of protein) and potassium bromate (1.07–3.17 µmol/g), glutathione (1.15–3.45 µmol/g) under hydrothermal processing	Gluten	Determination of reversed‐phase HPLC (RE‐HPLC), SE‐HPLC, and baking test	Lagrain et al. (2006)
Potassium bromate (200 and 2,000 ppm), potassium iodate (200 and 300 ppm), and dithiothreitol (DTT) (200 and 400 ppm)	Gluten	Determination of free sulphydryl (SH), SE‐HPLC, and pasting profile (RVA)	Lagrain et al. (2007)
Cysteine (0.2%)	Gluten	Determination of SE‐HPLC, Surface hydrophobicity, FTIR, rheological characteristics, Protein solubility	Zhang et al. (2010)
Potassium iodate (28.9 or 10,000 ppm), potassium bromate (22.5 or 10,000 ppm), l‐cysteine (98.0 or 10,000 ppm), glutathione (248.6 or 5,000 ppm), sodium metabisulfite (500 or 5,000 ppm) or N‐ethylmaleimide (101.2, 5,000 or 10,000 ppm)	Sugar‐snap cookie	Determination of time‐lapse photography, SEM, and protein extraction	Pareyt et al. (2010)
Cysteine (0.25–2 g/kg), N‐ethylmaleinimide (15 g/kg), and KIO_3_ (125 g/kg) under high pressure treatment	Gluten	Determination of rheological characteristics, protein solubility, SE‐HPLC	Schurer et al. (2007)
Cysteine (20 mg/kg)	Flour	Determination of rheological characteristics	Angioloni and Dalla Rosa (2007)
Cysteine (15–90 mg/kg)	Flour (wheat‐sorghum) (biscuit)	Determination of farinograph characteristics and baking test	Elkhalifa and El‐Tinay (2002)
Potassium bromate (20 and 40 ppm), ascorbic acid (100 and 200 ppm) and potassium iodate (20 and 40 ppm), reducing agents (potassium metabisulphite (100 and 200 ppm), and cysteine hydrochloride (50 and 100 ppm)	Flour	Determination of rheological characteristics and baking test	Indrani and Venkateswara Rao (2006)
l‐cysteine hydrochloride monohydrate (0.6 × 10^−2^–6.0 × 10^−2^% w/w), glutathione (6.0 × 10^−2^–18.0 × 10^−2^% w/w), l‐ascorbic acid (2.0 × 10^−2^–18.0 × 10^−2^% w/w l‐ascorbic acid in combination with l‐cysteine hydrochloride monohydrate, inactivated dry yeast (6.0 × 10^−2^% w/w), l‐threonine, l‐tryptophan); l‐threonine (1.2 × 10^−2^–18.0 × 10^−2^% w/w), l‐tryptophan (1.4 × 10^−2^–18.0 × 10^−2^% w/w)	Flour	Determination of rheological characteristics and baking properties	Pečivová et al. (2011)
KBrO3 and 20 amino acids (namely alanine, arginine, asparagine, aspartic acid, cystine, glutamine, glutamic acid, glycine, histidine, isoleucine, leucine, lysine, methionine, phenylalanine, proline, serine, tryptophan, threonine, tyrosine, and valine), and enzyme hydrolyzed gluten (1%)	Frozen dough bread	Determination of mixogram study, baking test, crust, and crumb color	Koh et al. (2005)
l‐cysteine hydrochloride, glutathione (50, 75, and 100 ppm)	Flour	Determination of gluten extraction and baking test	Kumar et al. (2014)
Cysteine (0%–0.75%)	Flour	Determination of rheological characteristics	Lambert and Kokini (2001)
NaCl (3 g), NaHCO_3_ (1.5 g), NH_4_HCO_3_ (3 g)	Biscuit Flour	Determination of rheological characteristics and baking test	Manohar and Rao (1997)
l‐cysteine (10%)	Flour	Determination of rheological characteristics and baking test	Stoica et al. (2010)
l‐cysteine (0%–2%) under extrusion process	Flour	Determination of rheological characteristics, electron paramagnetic resonance analysis, SEM, sulfhydryl‐disulfide content, SDS‐PAGE	Koh et al. (1996)
l‐cysteine (0.1 M or 0.01 M)	Flour	Determination of rheological characteristics, FTIR, and SEM	Yuno‐Ohta et al. (2009)
Glutathione, KBrO_3_, ascorbic acid, l‐threoascorbic acid, d‐erythreoascorbic acid (10–100 ppm)	Flour	Determination of rheological characteristics	Dong and Hoseney (1995)
Dithiothreitol (DTT), KIO_3_, KBrO_3_, H_2_O_2_ (0–200 μg/ml)	Flour	Determination of rheological characteristics	Bekes et al. (1994)
Sodium meta‐bisulfite (SMS) (360 mg/kg flour)	Biscuit flour	Determination of rheological characteristics	Pedersen et al. (2005)
NAD(P)H (0.02, 0.04, 0.06, 0.08, and 0.10 mM*)*	Flour	Determination of phosphodiesterase activity assay, free sulfhydryl level, protein extractability by SE‐HPLC	Joye et al. (2002)
Glutathione (1 μmol/g)		Determination of farinograph experiment	Kuninori and Sullivan (1968)
Ascorbic acid and [^35^S] GSH (1.85 × 10^6^ Bq) based on initial activity (125 ppm)	Flour	Determination of RP‐HPLC, GPC, and amino acid sequence	Koehler (2003)
Ascorbic acid and superoxide anion radical ($O_{2}^{‐}$)	Flour	Determination of rheological characteristics	Nakamura and Kurata (1997)
Ascorbic acid (30 ppm)	Gluten	Determination of farinograph and extensograph properties and baking test	Dagdelen and Gocmen (2007)
Ascorbic acid (10 ppm)	Flour	Determination of rheological characteristics, Zeleny sedimentation value, and baking test	Hrušková and Novotná (2003)
N‐ethylmaleimide (245 ppm), potassium iodate (70 ppm), and glutathione (100 ppm)	Pasta Flour	Determination of SE‐HPLC, RP‐HPLC, kinetics of gluten protein reactions	Bruneel, Lagrain, Brijs, and Delcour (2011)
l‐ascorbic acid, l‐cysteine hydrochloride, and sodium bisulfite (0–200 ppm)	Flour	Determination of protein solubility, SDS‐PAGE	Aminlari and Majzoobi (2002)
Water‐soluble α‐glucosyl rutin (G‐rutin) (200 – 1,000 ppm) l‐ascorbic acid (50 ppm)	Flour	Determination of farinograph and extensograph properties, baking test (loaf volume, color), and SEM	Morita et al. (1996)
Ozone (1,500 ppm for 2, 4.5, 9, and 18 min)	Flour	Determination of protein extraction, SE‐HPLC, and bread quality	Sandhu et al. (2011)
Ascorbic acid	Gluten	Determination of ascorbate oxidase activity, GSH‐DH, GSH, and CSH	Grosch and Wieser (1999)
KBrO_3_ (0–30 ppm)	Flour	Determination of SE‐HPLC, protein extraction characteristics	Panozzo et al. (1994)
Azodicarbonamide, ascorbic acid, boromate, l‐cysteine (15–150 ppm)	Flour	Determination of rheological measurement and baking test	Yamada and Preston (1994)
Bromate levels (0–250 ppm)	Flour	Determination of rheological characteristics and baking test	Preston and Dexter (1994)
KBrO_3_, KIO_3_, H_2_O_2_ (0–500 ppm)	Flour	Determination of SDS‐PAGE, Free sulfhydryl content, SE‐HPLC	Veraverbeke et al. (2000)
KBrO_3_ (25 ppm)	Flour	Determination of sulfhydryl and gas chromatographic determination of KBrO_3_ residue	Andrews et al. (1995)
KIO_3_ (0–10 mg/300 g)	Flour	Determination of rheological characteristics and baking test	Kohajdová and Karovičová (2010)
KIO_3_ (0–21 ppm)	Flour	Determination of rheological characteristics and baking test	Špačková et al. (2001)
Azodicarbonamide (45 mg/kg of flour)	Flour	Determination of semicarbazide formation	Noonan et al. (2008)
Chlorination up to pH 4.3 and 4.8	Flour	Determination of RP‐HPLC of gliadins, DSC of gliadin fraction, hydrophobicity changes of gliadins by fluorescence spectroscopy	Sinha, Yamamoto, and Ng (1997)
Cysteine (200 and 300 ppm), H_2_O_2_ (0.05%, 0.1%, and 0.2%)	Flour	Determination of rheological characteristics, baking test, and DSC	Thomasson et al. (1995)
Chlorine	Flour	Determination of protein extraction and SDS‐PAGE	Duviau et al. (1996)
Hydrogen peroxide (H_2_O_2_) (1.09–2.32 mmol/g of flour)	Flour	Determination of rheological characteristics, effect of hydrogen peroxide produced by glucose oxidase, effect of catalase on dough rheology	Liao et al. (1998)
Ascorbic acid, l‐cysteine, azodicarbonamide, calcium peroxide (6 and 12 ppm)	Flour	Determination of dynamic rheological characteristics	Miller and Hoseney (1999)
Ozone (0.06 L/min for 10 and 36 min)	Flour	Determination of mixograph characteristics, TPA, Size SE‐HPLC, and baking test	Chittrakorn (2008)
Ascorbic acid, potassium bromate or glutathione	Gluten	Determination of gluten extractability in SDS solution, SDS‐PAGE, farinograph and mixograph mixing tests, SS/SH content of flours, and glutens	Hayta and Schofield (2004)
Acetic acid + KIO_3_ + N‐ethylmaleimide (NEMI)	Gluten	Determination of SDS‐PAGE, dough mixing property	Sievert, Sapirstein, and Bushuk (1991)
Iodate, bromate, and N‐ethylmaleimide (NEMI) (300 μeq)	Flour	Determination of gluten extractability, RE‐HPLC	Tsen, (1969)
2‐Mercapto ethanol, N‐ethylmaleimide (NEMI), mercuric chloride, silver nitrate, sodium chloride, urea alone, and urea + ascorbic acid, and 0.07 N acetic acid (0–632 mg)	Gluten	Determination of farinograph characteristics	Zentner (1968)
Ascorbic acid (5, 50, and 500 ppm) and potassium bromate (3, 30, and 300 ppm)	Flour	Determination of rheological characteristics	Wikström and Eliasson (1998)

### 1.1 Reducing agents

Reducing agents weaken the gluten network through cleaving the disulfide bonds between gliadin, gluten, and glutenin proteins, promoting SH/SS interchange reactions in strong flour, and reducing the average molecular weight of glutenin protein aggregates (Fernandes & Ramos, 2004; Lagrain et al., 2006). SS bonds are covalent bonds with 205 kJ/mol energy. These bonds cannot typically be broken at room temperature unless with mechanical stress (sonication, high‐speed mixing), high temperature (baking), and chemical reactions via reducing agents in order to form reactive thiol radicals (Wieser, 2012). This mechanism reduces the dough strength, resistance to deformation, elastic and viscous behavior, and mixing time and tolerance to mixing, and the solubilization of wheat proteins, shortens the proofing period, prevents the formation of glutenin polymer (Schurer et al., 2007), and improves the machinability of dough and its handling features for high protein or strong dough (Angioloni & Dalla Rosa, 2007; Bekes et al., 1991; Joye et al., 2009a, 2009b). In contrast, machinability, extensibility, and adhesiveness are increased. l‐cysteine or l‐cysteine hydrochloride monohydrate, glutathione or inactivated dry yeast (chemically glutathione) and sodium metabisulfite, sodium sulfite, mercaptoethanol and dithiothreitol (DTT), ascorbic acid, and malt flour are among the reducing agents.

#### 1.1.1 l‐Cysteine

Gluten proteins both comprise a few cysteine residues (~2% of total amino acid composition and 20–25 μmol/g flour with a protein content of 10%–12%) and have a central part regarding the structure and functionality of gluten proteins (Wieser, 2007). Even under mild oxidative conditions, cysteine is easily converted to cystine; on the contrary, cystine is readily reduced to cysteine through the use of reducing agents (reaction 1) (Wieser, 2012):

(1) $${\text{2P}\;‐\;\text{CH}}_{2}\;‐\;\text{SH}\underset{+2H}{\overset{‐2H}{⟷}}{\text{DP}\;‐\;\text{CH}}_{2}{\;\text{‐ S ‐ S ‐ CH}}_{2}\;‐\;P$$

Even though l‐cysteine naturally exists in wheat flour, it can be commonly and extensively employed as food additive with GRAS status (Joye et al., 2009b) as a reducing agent. In l‐cysteine, the thiol group cleaves the disulfide bonds between proteins (‐SH) and weakens the gluten network (Aminlari & Majzoobi, 2002; Angioloni & Dalla Rosa, 2007; Min et al., 2008) possibly facilitating the enzymatic hydrolysis and effectively increasing the peptides with lower molecular weights. Most commonly, cysteine is utilized as hydrochloride (l‐cysteine hydrochloride) owing to its higher solubility and reaction at low concentrations varying from 10 to 90 ppm; nonetheless, it is normally added at 20 to 30 ppm during mixing (Angioloni & Dalla Rosa, 2007; Elkhalifa & El‐Tinay, 2002; Indrani & Venkateswara Rao, 2006; Pečivová et al., 2011). According to the results of Zhang et al. (2010) and Aminlari and Majzoobi (2002) upon supplementation with cysteine, the gluten MW decreased and the intensity of bands lower than 6.5 kDa increased (Aminlari & Majzoobi, 2002). This induced an increase in gluten protein solubility at isoelectric point and other pH values (Zhang et al., 2010). Based on the FTIR data attained by Mejri et al. (2005), β‐turn and β‐sheet increased, whereas α‐helices were reduced. Nonetheless, Zhang et al. (2010) observed a significant increase in β‐sheet, a minor improvement in α‐helices, the disappearance of extended structures (1,622 cm^−1^), and an increase in the intensity of OH stretching, ascribed to major structural changes caused by dissociated aggregation. Surface hydrophobicity decreased owing to the exposure of polar groups in polypeptide chains to the surface of gluten network and the breaking of intra‐ and interchain disulfide bonds in wheat gluten, hence the greater hydration capacity. Cysteine addition ameliorated extensibility, machinability, and adhesiveness and reduced the elastic (*G*′) and viscous (*G*″) characteristics, tolerance to mixing, and mixing time (Angioloni & Dalla Rosa, 2007; Indrani & Venkateswara Rao, 2006; Koh et al., 2005; Kumar et al., 2014; Lambert & Kokini, 2001; Manohar & Rao, 1997; Miller & Hoseney, 2008; Stoica et al., 2010; Yuno‐Ohta et al., 2009; Zhang et al., 2010) mainly because of (a) breaking of cross‐links and depolymerization (reactions 2 and 3), (b) increased sulphydryl (SH)–disulfide (SS) interchange reactions, (c) progressive water hydration capacity as a result of conformational changes, and (d) reduced surface hydrophobicity; the same as GSH, CSH further shows radical scavenging activity, thereby preventing the formation of dityrosine bonds and intra and/or intermolecular disulfide (SS) bonds between glutenin polymers (Angioloni & Dalla Rosa, 2007; Holler & Hopkins, 1989; Joye et al., 2009b; Koh et al., 1996, 2005; Kumar et al., 2014; Lagrain et al., 2006, 2007; Reinbold et al., 2008; Tilley et al., 2001; Zhang et al., 2010).

(2) CSH+PSSP→PSH+CSSP

(3) CSH+CSSP→PSH+CSSC

#### 1.1.2 Inactivated dry yeast (chemically named glutathione)

**FIGURE 2:**
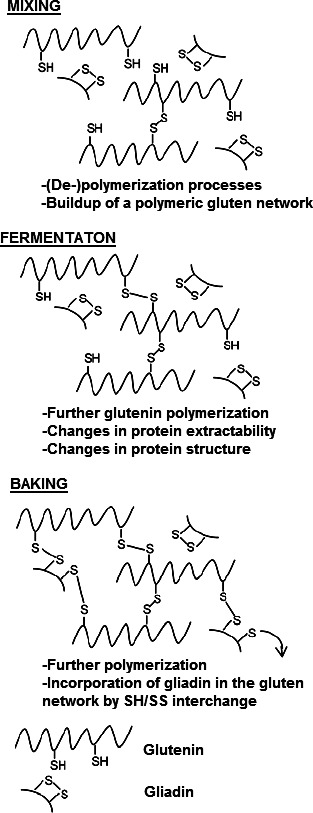
Three competitive reactions during breadmaking that impact on oxidation reactions. Disulfide (SS) containing proteins and sulfhydryl (SH) group containing proteins represented by PSSP and PSH, respectively (Joye, Shang, Brijs, & Delcour, 2010)

As a tripeptide (g‐glutamylcysteinylglycine), glutathione or inactivated dry yeast is composed of amino acid cysteine, glutamate, and glycine. At low amounts, this tripeptide is endogenous in the wheat germ, and it is a metabolic by‐product of yeast occurring at higher amounts in dry yeasts, hence utilized as an alternative GSH source (Joye et al., 2009b; Pyler & Gorton, 2009). Regarding to Li, Bollecker, et al. (2004) and Li, Tsiami, et al. (2004), the total free and protein‐bound glutathione compounds were estimated about 358 ± 51 and 190 ± 17 nmol/g, respectively, in the 36 wheat varieties. The existence of sulfhydryl (‐SH) group on the amino acid cysteine results in glutathione acting as a reducing agent readily oxidized to either GSSG or protein‐bound glutathione (PSSG). In the presence of dehydroascorbic acid (DHAA), glutathione dehydrogenase further catalyzes the oxidation of GSH to GSSG, thereby impacting the breadmaking process (Joye et al., 2009b). Endogenous GSH also contributes to the formation of SS bonds with single LMW‐GS, hence preventing their participation in gluten SS cross‐linking (Li, Bollecker, et al., 2004; Li, Tsiami, et al., 2004). During storage time, the decrease in the total amount of glutathione (GSH, oxidized glutathione (GSSG) and PSSG) led to the increased breadmaking behavior of wheat flour (Koehler, 2003a; Li, Bollecker, et al., 2004; Li, Tsiami, et al., 2004; Schofield & Chen, 1995). Glutathione is FDA‐approved (Fort, 2016; Yano, 2010), yet it cannot be added in large amounts because of its relatively high cost and dough stickiness. The existence of glutathione in wheat dough has been considered to augment extensibility, enhance the dough development, and reduce the elasticity, dehydration, crumb texture, and loaf volume of dough and bakery products (Dong & Hoseney, 1995; Every et al., 2006; Koh et al., 1996; Tilley et al., 2001). GSH is more highly incorporated in doughs compared with flour water suspensions. They proposed that mixing ensured SH/SS interchange even through augmenting the reaction between SH groups and SS groups (such as GSH) or via facilitating the production of glutathione and protein thiol radicals in the presence of oxygen; however, the relative rates of interchange and incorporation relatively depended on reactants having higher amounts in dough compared to suspensions (Figure 2) (Dong & Hoseney, 1995; Every et al., 2006; Koh et al., 1996; Tilley et al., 2001). Due to their high mobility, SH groups with low molecular weights (such as CSH and GSH) preferentially react with GSH for the formation of stable SS bonds. Grosch and Wieser (1999) reported that endogenous GSH preferentially reacted with the intermolecular SS bonds in charge of the covalent LMW‐GS bonds (reaction 4). In glutenin polymers, the breaking of interchain SS bonds reduced the average molecular weight of gluten proteins (Every et al., 2000). On the other hand, GSH probably cleaves some SS bonds in the proteins and generates new SH groups, thereby elevating the SS/SH interchange reactions. GSSG is also capable of binding to proteins through the use of SS bonds, causing PSSG (reaction 5). The generated GSH is able to react with the interchain SS bonds in the glutenin polymers. Nevertheless, the impact of dough softening is lower compared to GSH addition, which is owing to the consumption of half the molecules in the initial reaction between PSH and GSSG (Hahn et al., ; Every et al., 2000). Also, Lagrain et al. (2007) showed that through increasing the level of free SH groups, dough supplementation with GSH raised the degree of covalent gliadin–glutenin cross‐linking during baking.

(4) GSH+PSSP→GSSP+PSH

(5) GSSG+PSH→GSH+PSSG

According to Lagrain et al. (2006); Lagrain et al. (2007); Pareyt et al. (2010), low glutathione amounts did not significantly change the bread volume; however, high concentrations of reducing agents (10,000 mg/kg on a flour base) significantly reduced the diameter and set time of sugar‐snap cookie. Adding large amounts of reducing agents increased the intrinsic break strength of the cookie material, probably caused by more pronounced gliadin–glutenin cross‐linking (Pareyt et al., 2010). On the contrary, similar levels of GSH (as redox agent) significantly impacted the elasticity of the bread doughs (Bekes et al., 1994; Dong & Hoseney, 1995). Further studies showed that loaf volume and crumb texture were negatively correlated with the GSH level, and high levels of oxidizing agents delayed the setting and reduced the intrinsic break strength of the cookie (Every et al., 2006; Manohar & Rao, 1997; Pareyt et al., 2010; Pedersen et al., 2005). In the breadmaking process, glutathione concentration affected the extractabilities of *γ*‐gliadin more than α‐gliadin; however, it did not impact the extractability of *ω*‐gliadin due to the paucity of cysteine residues (Lagrain et al., 2006, 2007; Pareyt et al., 2010); additionally, B/C‐LMWGSs were more influenced compared with D‐LMWGSs and HMW‐GSs. During baking, the disulfide gliadin–glutenin cross‐linking increased in the presence of glutathione and other reducing agents (Lagrain et al., 2007), attributable to the SS bonds formed between glutenin and gliadin, indicating a SH/SS exchange mechanism.

#### 1.1.3 Sodium metabisulfite

Typically utilized as a biscuit/pastry dough‐softening agent in concentrations of around 10–200 mg/kg flour, this reducing agent decreases energy and water requirements upon mixing, elevates the dough extensibility, and enhances the shape of the final product. In comparison to cysteine, sodium metabisulfite is less expensive, but the former has been proven more effective and acceptable as a food additive because it is a naturally occurring amino acid (Joye et al., 2009b). Through water, the metabisulfite ion ($S_{2}O_{5}^{2‐}$) is hydrolyzed into bisulfite (${\text{HSO}}_{3}^{‐}$), reacting with protein SS groups via interchange, releasing an SH group on one protein yet freeing a thiosulfate ester on the other (reactions 6 and 7). To generate a free SH group on the protein and a sulfate ion (${\text{SO}}_{4}^{2‐}$) (reaction 8), this ester is hydrolyzed by water. These residues prevent the renewal of the formation of disulfide bonds (Fort, 2016).

(6) $$S_{2}O_{5}^{2‐}+H_{2}O\to {\text{HSO}}_{3}^{‐}$$

(7) $${\text{HSO}}_{3}^{‐}+\text{PSSP}\to {\text{PSSO}}_{3}^{‐}+\text{PSH}$$

(8) $${\text{PSSO}}_{3}^{‐}+H_{2}O\to \text{PSH}+{\text{SO}}_{4}^{2‐}+H^{+}$$

Unpleasant aftertaste of the final products and the destruction of the vitamin thiamine are the main downsides of sodium metabisulfite; therefore, not more than 10 ppm is to be employed in the final product. Metabisulfite ion is consumed along with oxidizing improvers such as sodium metabisulfite, bromate and ascorbic acid, and cysteine for the so‐called activated dough development as an alternative to high‐speed mechanical dough development (Fitchett & Frazier, 1986).

#### 1.1.4 NAD(P)(H)

Nicotinamide adenine (phosphate) dinucleotides (NADH and NADPH) were shown as direct or indirect bread enhancing agents through influencing gluten cross‐linking during breadmaking (reactions 9 and 10) (Joye et al., 2009b). NADH can act as an alternative to NADPH; however, the efficiency of this reaction is to some extent low. The NADP‐dependent glutathione system mostly affects the SS bonds of gliadins and low molecular weight glutenin subunits (Joye et al., 2002). The amount of oxidized nicotinamide coenzyme (NAD^+^) in wheat leaves is about 200 ppm, whereas normally, the amount of NAD(P)H and NADP^+^ is tenfold lower (Chen et al., 2004). This positively influences the breadmaking process via either supplementation or increased in situ availability (Joye et al., 2002). The nicotinamide co‐enzymes are considered as cofactors for many redox enzymes, including NAD(P)‐dependent dehydrogenases and NAD(P)‐thioredoxin reductase, prosthetic groups of myriad oxidoreductases such as glutathione reductase (GR); these co‐enzymes take part in reducing flavin co‐enzymes existing in both decreased [NAD(P)H] and oxidized [NAD(P)‏] forms in all living organisms (Joye et al., 2009b). Nicotinamide co‐enzymes primarily exert their effects through enzymatic reaction systems. Glutaredoxin reductase, thioredoxin, and NAD(P)‐dependent dehydrogenase are enzymatic systems naturally existing in wheat, requiring NAD(P)(H) for catalytic activity. Theoretically, they are capable of impacting gluten and dough features during breadmaking and in regard to the quality of the final product (Joye et al., 2002). Tomlinson et al. (1988) found that longer fermentation periods affected the properties of the baked product. Their assumption was that a metabolite or a group of metabolites such as oxidized or reduced co‐enzymes NAD(P)(H) was generated by fermentation, affecting the gluten (Tomlinson et al., 1988); there exists no consensus as to whether NAD(P)H or NAD(P)‏ is the most effective form of breadmaking since both are involved in numerous enzymic reactions able to play major roles in regard to characteristics (Joye et al., 2009b). Joye et al. (2002) reported that adding NAD^+^ (600 ppm) barely influenced the cake and bread volume; nevertheless, supplementation with NADH (600 mg/kg) and NADPH (600 mg/kg) significantly lowered the loaf volumes of cakes and breads prepared with of high‐quality breadmaking flour. The thiol content of wheat flour increased after incubating the wheat flour with NADH and NADPH under anaerobic conditions while NAD(P)^+^ improved the extractability in the SDS‐containing medium associated with the protein of the strong breadmaking flour.

(9) $$\text{PSSP}+\text{NADPH}+H^{+}⇄\text{2 PSH}+{\text{NADP}}^{+}$$

(10) $$\text{GSSG}+\text{NADPH}+H^{+}⇄\text{2 GSH}+{\text{NADP}}^{+}$$

### 1.2 Oxidizing agents

**FIGURE 3:**
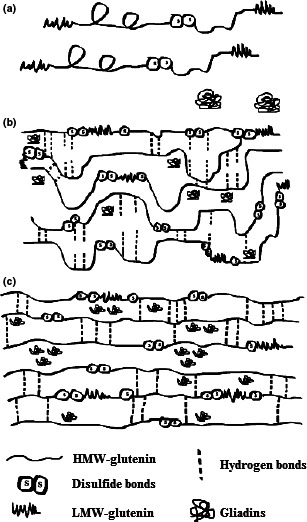
Schematic diagrams of the formation of gluten structure as a function of salt during hydration. (a) Unfolded native gluten structure in flour. (b) Gluten without oxidizing agent. (c) Gluten with oxidizing agent

Oxidizing agents facilitate the formation of SS bonds and minimize SH/SS interchange reactions, thereby strengthening the gluten network (Indrani & Venkateswara Rao, 2006; Lagrain et al., 2006, 2007, 2008; Miller & Hoseney, 2008; Vemulapalli et al., 1998). Meanwhile, via the oxidation of pigments, they exert a bleaching impact on flour (Wieser, 2012). Other oxidative reactions might further take place, including flour lipid oxidation and attachment of oxidative dimerization of feruloyl residues to flour pentosans (Figure 3) (Wieser, 2012).

Oxidizing agents enhance the carbon dioxide retention and dough handling features, improve the porosity of crumb in dough, ameliorate the quality of fresh bread, and increase the shelf‐life (Grausgruber et al., 2008; Hrušková & Novotná, 2003; Joye et al., 2009a; Lagrain et al., 2006; Morita et al., 1996). Cystine, l‐ascorbic acid, potassium bromate, l‐tryptophan and l‐threonine, potassium and calcium iodate, ozone, azodicarbonamide, and calcium peroxide were employed as oxidizing agents (Sandhu et al., 2011). The use of oxidants is generally approved in the United States; however, their use is prohibited in the European Union and only is permitted. In certain countries, ascorbic acid can be added to flour upon milling or incorporated into dough conditioner. Typically around 10–70 mg/kg flour, usable oxidant levels are dependent on the type of oxidant, flour, and the end product. For the development of mechanical dough, much higher amounts such as 100 mg/kg are employed.

#### 1.2.1 Ascorbic acid

Ascorbic acid (AH_2_) is a widely accepted additive in bread due to both function vitamin and antioxidant. As a reducing agent during mixing, AH_2_ weakens the gluten network where oxygen is scarce. Via the oxidation process in the presence of oxygen, it is also able to oxidize the dough characteristics and be converted into dehydroascorbic acid (Hrušková & Novotná, 2003; Joye et al., 2009a; Koehler, 2003a; Pečivová et al., 2011; Every et al., 2000). It has GRAS improver and is consumed (Joye et al., 2009a) in countries in which bromate is not allowed. On the other hand, compared with bromate, AH_2_ is less effective in improving loaf volume (Ranum, 1992). Small amounts of AH_2_ were proven to significantly ameliorate dough strength, increasing the loaf volume up to 20% higher than the control group. The amount of AH_2_ utilized in breadmaking varied between 20 and 150 ppm depending on the type and storage time of the flour, bread type, and breadmaking condition (Koehler, 2003a, 2003b). In dough, AH_2_ oxidation into DHAA by atmospheric oxygen was enhanced under both heat stable catalysts (heavy metal ions) and a heat‐labile enzyme ascorbic acid oxidase (AO) (Wieser, 2012).

**FIGURE 4:**
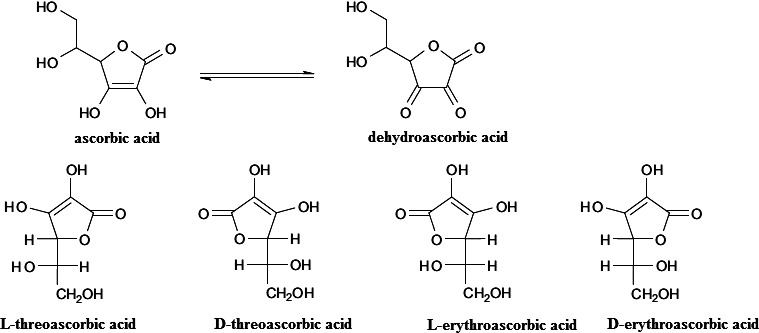
Different forms of ascorbic acid

AH_2_ comes in four forms, namely l‐threo‐AH_2_, d‐threo‐AH_2_, l‐erythro‐AH_2_, and d‐erythro‐AH_2_ (Figure 4). l‐threo‐AH_2_ is the one effective property of AH_2_ which improves the rheological features of dough, whereas d‐erythro‐AH_2_ and d‐threo‐AH_2_ are almost ineffective (Dong & Hoseney, 1995). Many mechanisms have been proposed to make use of the dough improving effect of AH_2_. The reaction is catalyzed enzymatically (reaction 11) and nonenzymatically (reaction 12) depending on the mixer speed and the level of oxygen mixed with dough (Wieser, 2012).

(11) $${\text{2L ‐ threo ‐ AH}}_{2}+O_{2}\overset{\text{AO}}{⟶}\text{2L ‐ threo ‐ DHA}+{\text{2H}}_{2}O$$

(12) $${\text{2 AH}}_{2}+1/{\text{2O}}_{2}\to \text{2DHA}+H_{2}O$$

**(A) Enzyme‐based reaction:** AH_2_ is rapidly oxidized into DHAA ( Grant & Sood, ) through atmospheric oxygen and endogenous ascorbate oxidase upon kneading and maturing (reaction 11). Acidic and basic AO enzymes both exist in wheat and have more affinity toward l‐AH_2_ compared with d‐AH_2_ (Every, 1999; Joye et al., 2009a). The enhancing effect of DHAA is based on the rapid removal of endogenous GSH (reaction 13); otherwise, GSH weakens the dough by SH/SS interchange with gluten proteins and is depolymerized through reacting with PSSP (reaction 14) (Wieser, 2012). l‐threo‐DA and dehydroreductic acid are the most optimal substrates for glutathione dehydrogenase (GSH‐DH) and AO and then two l(d)erythro‐AH_2_, whereas d‐threo‐AH_2_ is the unsuitable (Sarwin et al., 1993; Walther & Grosch, 1987; Wieser, 2012).

(13) $$\text{DHAA}+\text{2GSH}\overset{\text{GSH}\;‐\;\text{DH}}{⟶}\text{GSSG}+{\text{AH}}_{2}$$

(14) GSH+PSSP→GSSP+PSH

The reaction between DHAA and endogenous GSH generates its disulfide form, GSSG, via GSH‐DH (reaction 19) (Dong & Hoseney, 1995; Joye et al., 2009a; Kaid et al., 1997; Pečivová et al., 2011; Walther & Grosch, 1987). Subsequently, with the formation of protein‐bound glutathione (PSSG) and GSH, GSSG reacts with PSH (reaction 15) (Koehler, 2003a). It has been proposed that the amount of PSSG is improved during mixing. This influence is more effective through adding AH_2_, particularly concerning acid‐soluble glutenins. The rate of reaction between GSH and DHA by GSH‐DH (reaction 13) was much more rapid compared with the protein depolymerization caused by GSH reacting with PSSP (reaction 15) (Hahn & Grosch, 1998; Koehler, 2003a, 2003b). AH_2_ reduced the possible adverse effects of GSH on the dough system because of GSH conversion into GSSG and increased PSSP generation from PSH (reaction 15) (less depolymerization). These mechanisms are supported by the generation of mixed disulfides (PSSG) between GSSG and free SH groups of gluten proteins. Further corroborating this hypothesis, Huttner and Wieser (2001) reported the incorporation of S‐labeled GSH into Osborne fractions. With AH_2_ present, a significantly higher GSH proportion bound with the glutenins in comparison with no AH_2_. This research showed that the SH groups of gluten proteins forming PSSG in addition to AH_2_ can be selectively modified through adding S‐labeled GSH (reaction 15). Using this modification, the localization of these SH groups of the gluten proteins should become viable (Koehler, 2003a).

(15) GSSG+PSH→GSH+PSSG

Every (1999); Every et al. (1999); and Every et al. (2000) suggested that the direct oxidation of protein SH to SS bond proteins via DHAA was catalyzed by dehydroascorbate reductase along with AH_2_ production; however, Every et al. (2006) observed that direct oxidation between AH_2_ and SH proteins was caused by isomerase, which is naturally present in wheat (Joye et al., 2009a; Koehler, 2003a, 2003b). Besides GSH, other small water‐extractable SH compounds (CSH) are able to convert DHAA into AH_2_, when small GSH and GSH‐DH concentrations are inserted. Nonetheless, GSH‐DH does not directly catalyze the conversion of these small SH compounds. This indicates the emergence of a coupled oxidation/reduction reaction between GSSG and free CSH, catalyzed by GSH‐DH and other enzymes (reaction 16). Based on the foregoing hypothesis, this reaction entails CSH withdrawal from gluten proteins through the SH/SS interchange reaction (reactions 23 and 24) (Kaid et al., 1997; Kieffer et al., 1990).

(16) CSH+GSSG→GSH+GSSC

(17) CSH+PSSP→PSSC+PSH

(18) GSSC+PSH→PSSC+GSH

(19) CSSC+PSH→PSSC+CSH

Meanwhile, not only is GSH able to lower cystine (CSSC), but also thiols with low molecular weight (GSH and CSH) can react with the disulfide bonds of gluten proteins and induce depolymerization through cleaving the interprotein disulfide bonds of gluten proteins. To put it otherwise, reactions the same as GSH (reaction 15) could be assumed for CSH (reaction 17) (Huttner & Wieser, 2001). Intermolecular disulfide bonds stemming from LMW and the disulfide bond between HMW and LMW were cleaved via thiol/disulfide interchange and preferably attacked by GSH. Moreover, small proportions (around 5%) of intramolecular disulfide bonds of gluten proteins were further attacked. Huttner and Wieser (2001) carried out tests confirming that the interprotein disulfide bonds of gluten proteins were directly broken through the use of endogenous GSH after adding S‐labeled GSH. These findings imply that all the observed reactions regarding the action mode of AH_2_ improver have been experimentally proven except for the thiol/disulfide interchange of CSSC or GSSG with ‐SH groups of gluten proteins (reactions 15 and 19) (Koehler, 2003a, 2003b). Via dough mixing, cysteine residues belonging to subunits with low molecular weight are converted into protein–protein or glutathione–protein disulfides through thiol/disulfide interchange reactions (Koehler, 2003a, 2003b). Adding ascorbic acid to wheat‐fermented dough significantly optimized the proofing time and prolonged dough stability in samples with very short initial values (Hrušková & Novotná, 2003).

**(B) Free metal ion‐based reaction:** Adding both DHAA and AH_2_ improves the hardness of dough, but AH_2_ exerts more impact than DHAA (Nakamura & Kurata, 1997). AH_2_ is oxidized into DHA, a reaction catalyzable by free metal ions (Nakamura & Kurata, 1997). Reactive intermediates, namely superoxide anions, have been proposed to result from AH_2_ oxidation. Superoxide anions function as oxidative agents, breaking down proteins and generating SH radicals, which in turn produce intermolecular SS bonds. This assumption is corroborated through dough hardness assays where AH_2_ is added in the presence and absence of a superoxide scavenging system (Miyamoto & Nishimura, 2006; Nakamura & Kurata, 1997). The reassociation of SH radicals form intermolecular SS bonds and improve the bread structure (Miyamoto & Nishimura, 2006).

Dityrosine cross‐link is another linkage produced by DHAA for cross‐linking during dough mixing and baking (Tilley et al., 2001). On the contrary, in other studies supported by Rodriguez‐Mateos et al. (2006), through mixing and proofing, AH_2_ prevented the formation of dityrosine but had no effect on the final dityrosine level in bread. In the absence of molecular oxygen, AH_2_ (as an antioxidant) is not able to convert into DHAA, but it could scavenge radicals, thereby inhibiting the formation of dityrosine (Hanft & Koehler, 2005; Joye et al., 2009b; Pena et al., 2006).

#### 1.2.2 Halogenates

The halogenates employed in breadmaking are chlorate, iodate, and bromate as their potassium salts. The influence of halogenates is associated with the oxidation of free SH groups in dough, augmenting the gluten strength. In contrast to ascorbic acid, halogenates do not require molecular oxygen to ensure their improving effect. The major difference between bromate and iodate is the slow action of the former and the fast action of the latter (Wieser, 2012).

#### 1.2.3 Potassium bromate

As an oxidant and bleaching agent, potassium bromate (KBrO_3_) is commonly utilized in commercial breadmaking for a relatively long time and at high oven temperatures (25 min at 250°C) (Kaya & Topaktaş, 2007; Sandhu et al., 2011). Potassium bromate is a slow‐acting oxidizer decomposing into a stable potassium bromide (KBr); nonetheless, this conversion is particularly active during the latter steps of fermentation, probably owing to the reduced pH which enhances the strength of gluten during baking (Gandikota & MacRitchie, 2005; Joye et al., 2009a; Lagrain et al., 2007; Panozzo et al., 1994; Sandhu et al., 2011; Tenido, 2003). The optimum addition level varies from 10 to 50 ppm based on the flour (Dong & Hoseney, 1995; Joye et al., 2009a; Wieser, 2012; Yamada & Preston, 1994), but failure in the conversion of ${\text{BrO}}_{3}^{‐}$ to Br^−^ ensues residual potassium bromate in the bread. Bromate is further employed in combination with azodicarbonamide (ADA). Nakamura et al. (2006) still observed residual bromate in the bread crust when 9 ppm potassium bromate was added without other additives. Residual potassium bromate has been regarded as source of oxidative damage (Kasai et al., 1987; Sandhu et al., 2011) and detrimental to health and safety (Crosby et al., 2000; Joye et al., 2009a; Kurokawa et al., 1990; Parsons & Chipman, 2000; Sandhu et al., 2011; Shiao et al., 2002; Umemura et al., 1998). Potassium bromate is considered as a carcinogen, hence the significant reduction in its consumption in the breadmaking industry (Dupuis, 1997; Joye et al., 2009a; Kurokawa et al., 1990; Ranum, 1992; Sandhu et al., 2011). In the United States, very low amounts of bromate are still being legally utilized (Himata et al., 1997; Joye et al., 2009a; Shi et al., 2006). Basically, potassium bromate has no effect during mixing, and approximately half of the added bromate remains after mixing and a 4‐hr rest. During fermentation and baking, bromate influences the rheology of dough. Because at lower pH, bromate has a more rapid reaction, its influence is accelerated upon fermentation (Wieser, 2012).

Depending on the concentration of the added bromate, flour properties and process conditions (time and temperature) (Gandikota & MacRitchie, 2005; Lagrain et al., 2007) are capable of strengthening the doughs and improving dough features such as: (a) expansion, (b) loaf volume, (c) crumb structure and texture, and (d) rheological behaviors of the gluten matrix surrounding the gas cells for the prevention of gas cell rupture, particularly during the latter stages of proofing and baking (Joye et al., 2009a). Many studies found that the oxidative characteristic of bromate was associated with (a) oxidation of free SH groups of peptides with low molecular weights (such as GSH) (Dong & Hoseney, 1995; Hayta & Schofield, 2004; Schropp et al., 1995; Veraverbeke et al., 2000a). This creates small SS compounds and bromide (Br^−^), thereby prohibiting the participation of SH/SS interchange reactions of small peptides composed of at least one SH group and preventing depolymerization of gluten aggregates (Joye et al., 2009a); and (b) the inhibition of the activity of proteolytic enzymes in the wheat flour: the initial rate‐limiting reduction of bromate to bromite by thiol groups proceeds slowly for bromate (reaction 20). Afterward, the reduction of bromite to bromide and oxidation of SH to SS groups occur as a fast stage (reaction 21) (Wieser, 2012).

(20) $$\begin{array}{c} \text{Slow}\\ {\text{BrO}}_{3}^{‐}+\text{2PSH}\to {\text{BrO}}_{2}^{‐}+\text{PSSP}+H_{2}O \end{array}$$

(21) $$\begin{array}{c} \text{Fast}\\ {\text{BrO}}_{2}^{‐}+\text{4PSH}\to {\text{Br}}^{‐}+\text{2PSSP}+{\text{2H}}_{2}O \end{array}$$

During baking, bromate is able to reduce gliadin–glutenin cross‐linking as a result of the reduction in the total amount of SH groups available for cross‐linking (Lagrain et al., 2007). Andrews et al. (1995) reported no direct relationship between bromate addition and the reduced levels of free SH groups; however, Hanft and Koehler (2005) found a probable association between time and the formation of tyrosine cross‐links and potassium bromate as oxidant at high temperatures.

#### 1.2.4 Potassium iodate

Known as a fast‐acting oxidant (Kohajdová & Karovičová, 2010), potassium iodate is completely consumed during mixing, thereby limiting the oxidizing effect of iodate to the dough mixing process (Wieser, 2012). Because of its higher oxidation potential (${\text{IO}}_{3}^{‐}=1.0\text{9 V},\;{\text{BrO}}_{3}^{‐}=+0.\text{61 V}$) and faster action, fewer iodate equivalents are required compared with bromate for the purpose of achieving the optimal rheological properties of dough for breadmaking (Wieser, 2012). The optimum level of iodate addition was suggested to range from 10 to 20 ppm (Wieser, 2012). The action mode of iodate is ascribed to the reduction of iodate to iodite (reaction 22); subsequently, the iodide (reaction 23) by thiol groups proceeds rapidly and SH is oxidized into SS groups as a fast stage (Bürgi et al., 2001; Kohajdová & Karovičová, 2010; Špačková et al., 2001).

(22) $$\begin{array}{c} \text{Fast}\\ {\text{KIO}}_{3}^{‐}+\text{2PSH}\to {\text{KIO}}_{2}^{‐}+\text{PSSP}+H_{2}O \end{array}$$

(23) $$\begin{array}{c} \text{Fast}\\ {\text{KIO}}_{2}^{‐}+\text{4PSH}\to I^{‐}+\text{2PSSP}+{\text{2H}}_{2}O \end{array}$$

Similar to potassium bromate, potassium iodate increases the mean molecular size of the protein, ascribed to the oxidation of sulfhydryl groups of glutenin subunits with high and low molecular weight (Sandhu et al., 2011; Schropp et al., 1995; Veraverbeke et al., 2000a, 2000b). Adding potassium iodate to wheat flour reduced the time of dough development, prolonged the dough stability with a volume higher than the control sample (Kohajdová & Karovičová, 2010).

Iodate has been affirmed as GRAS, and its addition to dough counteracts iodide deficiencies in the diet; however, the wide use of iodates in the breadmaking industry might entail problems (Ranum, 1992) such as generating overoxidized dough and reducing the quality of loaf. Veraverbeke et al. (2000a, b) found that high iodate concentrations negatively affected the polymerization of high molecular weight glutenin subunits (HMW‐GS) during oxidation at pH 3.0. It seems that high iodate concentrations favor the oxidation of SH groups to higher sulfur oxidation states (Joye et al., 2009a); hence, reducing the degree of HMW‐GS cross‐linking. (Veraverbeke et al., 1999) reported that iodate augmented the extractability of glutenin during mixing and reduced the level of unextractable glutenin during fermentation. Lagrain et al. (2007) observed the increased extractability of α‐ and γ‐gliadins during baking.

#### 1.2.5 Chlorine dioxide and chlorine

As a bleaching agent and a weak gaseous oxidant, chlorine dioxide (ClO_2_) can be employed up to 2,500 ppm for breadmaking. Chlorinated flour is commonly prepared to bake cakes with enhanced qualities such as white crumb, high volume, good sensory behaviors, and uniform and fine grain. The enhancing mechanism is difficult to elucidate (Joye et al., 2009a; Thomasson et al., 1995); nonetheless, Tsen et al. (1971) suggested that in addition to the cleaving of intra‐ and intermolecular hydrogen bonds and the breaking of peptide bonds, aromatic amino acids were degraded and SH was oxidized to SS groups in chlorine treatment. Duviau et al. (1996); Joye et al. (2009a) showed that the free SH content of flour decreased, whereas SS bonds were barely impacted following chlorination. Compared with gliadin, glutenin proteins were less influenced by chlorination (Joye et al., 2009a). Cakes baked at the optimal chlorination concentration (about 1,100–2,300 ppm) do not collapse during baking, are more tender, and have a fine uniform grain, white crumb color, and enhanced symmetry and volume (Gough et al., 1978; Thomasson et al., 1995). Chlorinated flour is constantly generated through agitating flour with low protein content (less than 9%) in a stream of chlorine gas so as to form hypochlorous acid (HClO) and chlorine ion (Cl^−^). Hypochlorous acid is a weak acid dissociated into hypochlorite ion (ClO^−^) and hydrogen ion (H^+^) (Chittrakorn, 2008; Gough et al., 1978; Wei et al., 1985). Chlorination influenced gluten proteins and cleaved peptide bonds, thereby increasing the solubility of protein in water. Oxidized SH groups and decomposed aromatic amino acids were further observed during chlorine treatment (Chittrakorn, 2008; Gough et al., 1978; Kulp, 1972; Tsen et al., 1971; Wei et al., 1985). Kulp (1972) reported that upon oxidative and hydrolytic action, chlorine was able to break down high molecular weight proteins. Duviau et al. (1996) observed altered protein extractability and reduced SH groups in the flour treated by chlorine, while no changes were detected in SS bonds. Tsen et al. (1971) reported that the dissociation of intra‐ and intermolecular noncovalent bonds and the cleavage of peptide linkages might improve the extractability of protein. Duviau et al. (1996) revealed that the extractability of gluten proteins underwent significant changes, especially at pH 4.3 than to pH 4.8 upon chlorination. Gliadins sounded to be highly affected than glutenins. Furthermore, LMW proteins in ranged between 9 and 15 kDa were also substantially impacted. Since free ‐SH groups reduced slightly in chlorinated proteins, implying that SS bonds have not changed under chlorination, and their accessibility to thioredoxin and to dithiothreitol also maintained unchanged. Chlorinated gliadins represented slightly more *T_d_* values than unchlorinated gliadins, suggesting an enhancement in hydrophobicities of chlorinated protein. Moreover, Δ*H* values of gliadins from chlorinated flours showed lower than those from unchlorinated samples due to partial unfolding of gliadins and less energy was required to disrupt the ordered structure of gliadins through chlorination.

#### 1.2.6 Azodicarbonamide

Owing to its low cost, ADA powder is an alternative compound for bromate, iodate, and chlorine dioxide (Joye et al., 2009a; Ranum, 1992). The permissible amounts of ADA vary from 10 to 45 ppm (de la Calle & Anklam, 2005; Joye et al., 2009a; Wieser, 2012; Yamada & Preston, 1994). Even though ADA consumption as dough improver is prohibited in the European Union, it is utilized in many countries such as the USA and Canada (Noonan et al., 2008) and Brazil (Pereira et al., 2004).

ADA functions similarly to, even faster than, iodate, and it is converted to biurea and semicarbazide during baking (de la Calle & Anklam, 2005; Joye et al., 2009a; Pereira et al., 2004). Due to its carcinogenic or mutagenic effects, semicarbazide might entail health risks (Joye et al., 2009a; Noonan et al., 2008). The amount of semicarbazide can be particularly high in bread crust (de la Calle & Anklam, 2005; Joye et al., 2009a, 2009b) (reaction 24). The oxidative property of ADA is attributed to the initial rapid oxidization of free SH groups to disulfide bonds and then the stimulation for the production of dityrosine cross‐links in bread (Joye et al., 2009a, 2009b; Tilley et al., 2001).

(24) ‐N=N‐+2PSH→PSSP+‐ NH ‐ NH ‐

Its action in dough can be enhanced through increased resistance to extension, strengthened dough, cohesive, and dry dough able to tolerate high water absorption, ameliorated machinability, gas retention, and improved loaf texture and volume (reaction 24) (Joye et al., 2009a; Wieser, 2012; Yamada & Preston, 1994). At same iodate weight, mixing times are shortened and energy input in dough mixing is reduced (Wieser, 2012). ADA overtreatment is determined by gray and streaky crumb with poor volume, causing extensible and tight dough. This has to be countered in commercial improver mixer through mixing with other oxidants and reduced enzymes (Wieser, 2012).

#### 1.2.7 Molecular oxygen, peroxides, and ozone

Molecular oxygen (O_2_) is the natural maturing agent for oxidizing agent in breadmaking. The enhancing mechanisms of O_2_ have yet to be fully elucidated; however, dough mixing incorporates air in the gluten matrix, and molecular oxygen increases gluten cross‐linking and eliminates the SH groups. This in turn augments the resistance to extension and reduces extensibility. Oxygen further bleaches the dough (Joye et al., 2009a; Wieser, 2012; Xu, 2001). Compared with a nitrogen atmosphere, mixing dough under oxygen conditions produces stronger doughs with higher resistance and lower extensibility; on the other hand, the oxygen concentration is an important factor (Joye et al., 2009a; Xu, 2001). Regarding other oxidants, the effect of oxygen is highly dependent on the concentration (Xu, 2001). The significance of oxygen in regard to dough stability and volume was investigated with its elimination from the surrounding air during dough formation (Marston, 1986; Xu, 2001). According to the studies on flour water slurries, it can be assumed that oxygen omission from dough could be a physical phenomenon; however, the oxygen, quickly desorbed from hydrated starch granules and fibers (dough matrix), is able to improve the volume of dough during the baking process (Marston, 1986; Xu, 2001).

Peroxides like calcium peroxide (CP), benzoyl peroxide (BP), and acetone peroxide (AP) are possibly added at concentrations between 6 and 15 mg/kg and 75 mg/kg and 20–35 mg/kg, respectively (Joye et al., 2009a; Wieser, 2012). Based on the WHO reports (2005), peroxide does not have a carcinogenic effect in humans. As a highly fast‐acting oxidant, AP quickly reduces the SH level of dough and increases its resistance. Contrary to other improvers, AP acts in the dry flour within 24 hr, representing a good tolerance to overtreatment but with poor storage features (Wieser, 2012). To obtain similar effects in dough, AP equivalents more than those of azodicarbonamide or iodate are required (Wieser, 2012). The common dose is approximately 25 mg/kg flour.

When solubilized in water, calcium peroxide releases hydrogen peroxide (H_2_O_2_) (reaction 25), which is an almost important active compound. The hydrogen peroxide portion is further released in dough via yeast or lactobacillus fermentation, affecting dough rheology because of radical cross‐linking reactions initiated by peroxidase which transforms hydrogen peroxide into free radicals (Liao et al., 1998). Subsequently, these free radicals are able to form a network among (a) CSH residues of protein, (b) tyrosine residues of gluten proteins (dityrosine cross‐links), and (c) ferulic acid and protein tyrosine residues. Three types of cross‐links have been evidenced by numerous research groups (Hanft & Koehler, 2005; Joye et al., 2009a; Rodriguez‐Mateos et al., 2006). Adding peroxide results in stronger and more elastic doughs, decreased dough stickiness, and enhanced machinability, handling, and water absorption (Liao et al., 1998; Miller & Hoseney, 1999; Tieckelmann & Steele, 1991). When calcium peroxide and AH_2_ are simultaneously inserted, AH_2_ oxidation to DHA is preceded by calcium peroxide, thereby increasing the effect of AH_2_ (Ranum, 1992). This material is typically mixed with enzyme‐active soy flour with a recommended dose range of 20–35 mg/kg.

(25) $${\text{CaO}}_{2}+H_{2}O\to \text{CaO}+H_{2}O_{2}$$

The influence of the superoxide anion radical ($O_{2}^{‐}$) on hardness of dough was investigated by using both $O_{2}^{‐}$ scavenging and $O_{2}^{‐}$ generating systems. Under $O_{2}^{‐}$ generating system condition (xanthine and xanthine oxidase), dough hardness enhanced, while upon $O_{2}^{‐}$ scavenging system (catalase and superoxide dismutase), dough hardness was same as the untreated protein (Nakamura & Kurata, 1997).

Ozone (O_3_) is a strong oxidative agent introduced as GRAS (Dubois et al., 2006). This agent is produced on site, eliminating the need for its storage. Moreover, ozone has a half‐life of 20–50 min and rapidly breaks down into molecular oxygen without leaving a residue. The foregoing upside makes ozone a possible safe alternative to such harmful chemical reagents as potassium bromate (Sandhu et al., 2011). Ozone is capable of oxidizing both organic and inorganic compounds and reacting with organic compounds by two pathways: free radicals or cyclo addition. Ozone is also able to react with multiple bonds or nucleophilic sites (N, O, P, S) of different organic compounds, including alcohols, aldehydes, carboxylic, ethers, acids, aromatic compounds, phenols, amine derivatives, carbohydrates, and amino acids, particularly cysteine, methionine, tryptophan, histidine, tyrosine, and cysteine. At neutral and basic pH, ozone highly reacts with both R groups and amine groups of amino acids, namely sulfur, alkyl, and aromatic or unsaturated heterocyclic groups, producing various types of products such as nitrate ions, aldehydes, ammonia, acids, condensation products, and aromatic aliphatic acids (Langlais et al., 1991; Mehlman & Borek, 1987). Yvin et al. (2005) observed that doughs produced by ozone‐treated flour had increased strength and reduced extensibility. Chittrakorn (2008) found that cakes baked with soft wheat flour treated with ozone (as oxidizing agent) presented softer texture and larger volume compared with those produced with chlorinated flour. In addition, increased ozonation time decreased the hardness of cakes. Sandhu et al. (2011) detected increased insoluble polymeric proteins owing to intermolecular disulfide bonds formed by the oxidation of sulfhydryl groups in the ozone‐treated flour. The specific volume of bread baked with ozone‐treated flour did not significantly differ from that produced with flour containing potassium bromate. Ibanoglu, 2002; İbanoǧlu (2001) examined hard and soft wheat grains tempered with ozonated water, which did not significantly change the rheological characteristics of flour. Earles, 2003; Chittrakorn (2008) observed that cakes baked with ozonated flour exhibited higher volumes compared with the control chlorinated flour.

#### 1.2.8 Amino acids

Adding amino acids both improved the organoleptic and nutritional qualities of final bakery products and enhanced its rheological features (Koh et al., 2005). Cysteine and aspartic acid addition at 1% concentration lowered the mixing tolerance of dough because of the disulfide interchange reaction and the reducing reaction of sulfhydryl (–SH) on disulfide cross‐link of gluten protein (Koh et al., 2005). Nevertheless, cystine has no free sulfhydryl, thereby not impacting the significant dough breakdown after the optimum mixing time (Koh et al., 2005). Glutamic acid augmented the mixing tolerance, whereas lysine, arginine, and histidine, as basic amino acids, delayed the optimum mixing time and showed a minor increase in the mixing tolerance (Koh et al., 2005). Aromatic amino acids phenylalanine, tyrosine, and tryptophan did not significantly affect the mixing characteristics of dough; however, Tilley et al. (2001) revealed that tyrosine elevated the mixing tolerance of flour owing to dityrosine cross‐linkage (Hanft & Koehler, 2005). Other such amino acids as asparagine, glycine, alanine, glutamine, isoleucine, threonine, serine, and valine marginally augmented the optimum mixing time and did significantly impact the dough mixing features (Koh et al., 2005). Addition of 1% cystine, methionine, tryptophan, and phenylalanine significantly increased the loaf volume in comparison to the control bread (*p* < .05). Certain amino acids, including glycine, lysine, and glutamic acid, were effective in improving the loaf volume (Zentner, 1961); on the other hand, Rubenthaler et al. (1963) reported that glutamic acid enhanced the loaf volume to some extent, glycine had the most adverse effect on the loaf volume, and lysine did not exert a significant impact. Koh et al. (2005) detected a significant decrease in the loaf volume via adding glycine, histidine, glutamic acid, isoleucine, arginine, lysine, and aspartic acid; however, the addition of cystine, methionine, tryptophan, and phenylalanine effectively reduced the loaf volume of bread baked with frozen dough. l‐tryptophan and l‐threonine were found to be the weakest oxidizing agent compared to other oxidant. The combination of l‐tryptophan or l‐threonine with l‐ascorbic acid had a stronger strengthening impact on the gluten network (Pečivová et al., 2011). Cystine and cysteine are another amino acids that affect dough properties which were discussed in cysteine section. Added at a concentration of around 20 ppm, l‐cystine (CSSC) improved the dough and bread properties (Yamada & Preston, 1994). The same as GSSG, CSSC had a more moderate impact on dough compared with its reduced form, GSH and CSH, respectively (Hahn & Grosch, 1998). CSSC addition might further improve the volume of loaf (Koh et al., 2005).

### 1.3 The effect of redox additives during heating treatment

**FIGURE 5:**
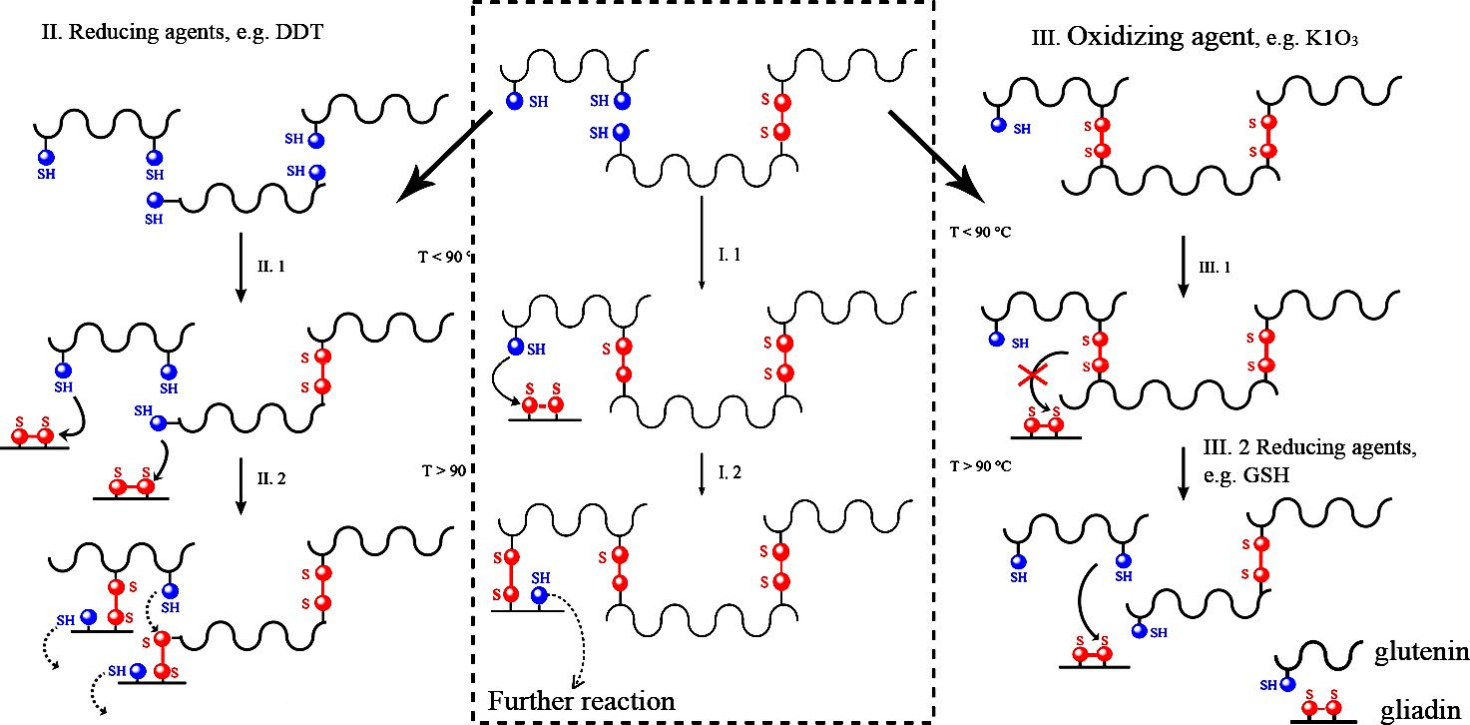
Model for gliadin–glutenin cross‐linking upon SH‐SS exchange reactions during hydrothermal treatment. (I.1): In the absence of additives, heating to 90°C leads to conformational changes exposing previously unavailable free SH groups and polymerization of glutenin with the oxidation of SH groups. (I.2) Glutenin is able to react to gliadin at temperatures exceeding 90°C via SH‐SS interchange and the produced free SH group can further react with either gliadin or glutenin. (II.1) Reducing agents depolymerize glutenin and increase the level of free SH groups, thereby (II.2) increasing gliadin–glutenin cross‐linking. (III.1) In the presence of an oxidizing agent reduces the level of free SH, thereby hindering glutenin linking and gliadin–glutenin cross‐linking above 90°C. (III.2) Subsequent addition of a SH containing agent can introduce new free SH groups in the gluten proteins and once induce gliadin–glutenin covalent cross‐linking (Lagrain et al., 2008)

Numerous studies have reported the effect of the presence and absence of redox additives (potassium bromate/iodate, ascorbic acid, glutathione, and dithiothreitol) on free SH and gliadin–glutenin association through the heating process (Figure 5) (Andrews et al., 1995; Grosch & Wieser, 1999; Hayta & Schofield, 2004; Lagrain et al., 2006; Lavelli, Guerrieri, & Cerletti, 1996; Yamada & Preston, 1992). Redox agents have diverse effects on the SDS extractability of gluten depending on the type of additive, the temperature, and quality of gluten from poor or good breadmaking flour. Ascorbic acid and potassium bromate/iodate, as oxidizing agents, improve the solubilization of insoluble protein and reduce RVA viscosities due to the depolymerization of gluten and reduction in gliadin–glutenin cross‐linking during heating‐mixing; however, glutathione, as a reducing agent, facilitates polymerization reactions through SH‐SS exchange reactions and reduction in protein extractability (Lagrain et al., 2006, 2008; Lambrecht, Rombouts, De Ketelaere & Delcour 2017; Lavelli et al. 1996).

## 2 CONCLUSION

Gluten proteins are known widely and abundant plant protein resources in nature. Chemical modification reactions involved reaction of gluten protein with reagents, which cause degradation, and aggregation, manipulating secondary and tertiary structure induced by interaction changes including ionic, hydrogen, and disulfide bonds, as well as hydrophobic and electrostatic interactions. They play an important role in determining the unique conformational functional and rheological properties of dough and baking quality of gluten. Hence, we tried to fulfill gaps of knowledge about various kind of chemical modification research, which modified gluten will be suitable in different application of the food and nonfood products. Additional point is that a better understanding of the correlations between structure and functionality of gluten proteins when undergoing chemical modification by using reagents.

## CONFLICT OF INTEREST

The authors declare that they do not have any conflict of interest.

## ETHICAL APPROVAL

Human and animal testing is unnecessary in this study.

## Data Availability

Research data are not shared.
